# Impact of poverty and adversity on perceived family support in adolescence: findings from the UK Millennium Cohort Study

**DOI:** 10.1007/s00787-024-02389-8

**Published:** 2024-02-14

**Authors:** Nicholas Kofi Adjei, Kenisha Russell Jonsson, Viviane S. Straatmann, Gabriella Melis, Ruth McGovern, Eileen Kaner, Ingrid Wolfe, David C. Taylor-Robinson, the ORACLE consortium, Simon Barrett, Sarwar Tubah, Julia Forman, Raeena Hirve, Mary Bangisky, Harriet Boulding, Simon Hackett, Julia Fox-Rushby, Cassey Muir, Kedzior Sophie, Abigail Salmon

**Affiliations:** 1 Department of Public Health, Policy and Systems, University of Liverpool, Liverpool, UK; 2 School of Public Health and Community Medicine, Institute of Medicine, Gothenburg University, Göteborg, Sweden; 3 Department of Public Health Sciences, Stockholm University, Stockholm, Sweden; 4 Population Health Sciences Institute, Newcastle University, Newcastle, UK; 5 Department of Women and Children’s Health, King’s College London, London, UK

**Keywords:** Poverty, Adversity, Trajectory analysis, Adolescents, Emotional support

## Abstract

Emotional support from family members may have an important effect on adolescent health outcomes, and has been identified as a target for policy to protect against the impacts of poverty and other early life adversities. However, few studies have assessed the extent to which poverty and adversity themselves influence the nature of emotional support that parents can provide to adolescents. We, therefore, aimed to investigate the impact of trajectories of income poverty and family adversities, including parental mental ill health, alcohol misuse and domestic violence across childhood developmental stages on young people’s relationships with their families and perceived emotional support received. We analysed longitudinal data on 10,976 children from the nationally representative UK Millennium Cohort study. Exposure trajectories of poverty and family adversities were characterised using group-based multi-trajectory models (age 9 months–14 years). The outcomes were perceived emotional support and quality of family relationships, measured by the three-item Short Social Provisions Scale (SPS-3) and levels of parent–adolescent closeness and conflict, measured at age 14. ORs and 95% CIs were estimated using multivariable logistic regression models, adjusting for potential confounding factors. At age 14, the overall prevalence of low perceived emotional support was 13% (95% CI: 12, 14). Children of mothers with lower socioeconomic status (SES) were more likely to report low emotional support, with a clear social gradient (education—degree plus: 10.3% vs. no qualifications: 15.4%). Compared with children exposed to low levels of poverty and adversity, children in the persistent adversity trajectory groups experienced higher odds of low emotional support and low-quality parent–adolescent relationship; those exposed to both persistent poverty and poor parental mental health were particularly at increased risk of experiencing poor family relationships and low perceived emotional support (adjusted odds ratio 2·2; 95% CI 1·7–2·9). Low perceived emotional support and poor family relationships in adolescence are more prevalent among socially disadvantaged children and adolescents and those experiencing social adversity. Policies to improve levels of family support for UK adolescents should focus on improving modifiable determinants such as child poverty and family mental health.

**Supplementary Information:**

The online version contains supplementary material available at 10.1007/s00787-024-02389-8.

## Introduction

Over the past decade, an increasing number of studies have consistently linked social and emotional support to the psychological, personal and relational well-being of individuals across the life course [1, 2]. Among young people, perceived emotional support from family members (i.e., care, safety, advice and having someone to talk to), in particular, has been shown to be associated with outcomes such as school performance and academic attainment [3], and overall physical and mental health [4, 5].

The importance of family support in public policy has been increasingly recognised in recent years [6, 7], and it is now considered a central component of some national strategies aimed at improving outcomes for young people [8]. In the UK, for example, the recent Children’s Commissioner report [7] highlights that policy and practice should focus on building strong and supportive families to ensure that children and young people have the social and emotional support they need to achieve their full potential. The report also emphasises the potentially protective effect of quality family relationships and strong emotional support for children and young people experiencing social adversities [7].

However, prior research suggests that social and emotional support are unevenly distributed in families [9, 10]. A number of important structural factors, including poverty, may greatly impact the level and nature of emotional support that families are able to provide to young people [11]. Theories such as the family stress model suggest that disadvantaged circumstances can make it difficult for families to provide care and practical support, due to the impact of financial insecurity on parental mental wellbeing and family functioning [12]. Additionally, material deprivation can limit the time, resources and socially supportive networks that facilitate high-quality family support for young people [10]. Furthermore, children born into disadvantaged socioeconomic circumstances are more likely themselves to experience poor physical and mental health outcomes, which may further impact the quality of parent–child relationships [12, 13].

Very limited research, however, has moved beyond the single adversity paradigm to explore the impact of multiple family-related risk factors on the perceived level of family support experienced by young people and the quality of parent–child relationships (i.e., conflict and closeness) from childhood to adolescence [14]. According to the theory of syndemics, childhood adversities such as poverty and family mental health problems tend to cluster together and accumulate over time, leading to cascading negative outcomes over the lifecourse [15]. This current study, therefore, aimed to build on our previous work on the clustering of family adversities [16], by assessing the long-term impact of child poverty and family adversity on young people’s family relationships and perceived levels of emotional support.

## Methods

### Study setting and participants

We used data from the Millennium Cohort Study (MCS), a nationally representative contemporary cohort of children born in the UK between September 2000 and January 2002, and followed up through seven survey waves. The first information was collected when cohort members were around 9 months old, with subsequent follow-ups at 3, 5, 7, 11, 14, and 17 years of age. In this analysis we used data on singletons (i.e., not twin or other multiple pregnancies) from wave 1 (9 months) to wave 6 (14 years) since our outcomes of interest were only measured at age 14 years (see below). The respective numbers of participant at each wave were 18,552, 15,590, 15,246, 13,857, 13,287, and 11,726. At each wave, information was collected from the primary caregiver (usually the child’s mother) and partner respondents. The MCS oversampled children from Wales, Scotland and Northern Ireland, disadvantaged areas and, in England, areas with higher ethnic minority populations, by means of stratified clustering sampling design. Additional information on the survey design and sampling is detailed elsewhere [17].

### Exposures

The main exposures were the longitudinal trajectories of poverty and family adversity that we defined in our previous study [16]. Poverty was defined as household equivalised income of less than 60% of the national median income [16, 18]. Family adversity included measures of parental mental illness, domestic violence and abuse and alcohol use [16] (for full details see supplementary appendix, pp 1). Our previous study identified six adversity trajectory groups experienced by UK children from birth to age 14 years, established using a group-based multi trajectory modelling technique (details below) [16]. The low poverty and adversity group contains children with an overall low exposure to childhood family adversities. The persistent poverty group comprises children with a high likelihood of poverty throughout childhood. The persistent poor parental mental health group is mainly characterised by high rates of poor parental mental health over time. The persistent parental alcohol use and domestic violence groups comprise children exposed to parental alcohol use and domestic violence throughout childhood, respectively. Finally, the persistent poverty and poor parental mental health group contains children with high exposure to the co-occurrence of both persistent poverty and poor parental mental health throughout childhood [16].

### Outcomes

The main outcomes were perceived emotional support and other measures of the quality of parent–adolescent relationship reported by adolescents at age 14 years. Perceived emotional support was measured using the three-item Short Social Provisions Scale (SPS-3). The SPS-3 consists of three items assessing levels of emotional support: ‘*I have family and friends that help me feel safe, secure, and happy*’; ‘*There is someone I trust whom I would turn to for advice if I were having problems*’; ‘*There is no one I feel close to*’ (reversed ordered). Response categories ranged from 1 ‘*Very true*’, 2 ‘*Partly true*’, and 3 ‘*Not true at all*’. The individual items were summed to create a score that ranged from 3 to 9, with higher values indicating higher emotional support. A cut-off score of −1.25 below the normed mean score was used to define whether an adolescent had low perceived emotional support (coded as 1) or high emotional support (coded as 0).

Parent–adolescent relationship was measured using two questions related to frequency of quarrelling (conflict) and closeness, self-reported by adolescents and their parents. To assess frequency of quarrelling, we used questions on whether adolescents have occasional quarrels with their parents. Response categories ranged from 1 ‘*Hardly ever*’ to 5 ‘*Most days*’. For closeness, adolescents were asked how close they are to their parents. Response categories ranged from 1 ‘*Not very close’* to 4 ‘*Extremely close’* (reversed ordered). Parents were also asked the same questions regarding their children for both dimensions separately. Conflict and closeness dimensions have often been used to describe parent–adolescent relationships [19].

### Potential confounders

**Fig. 1:**
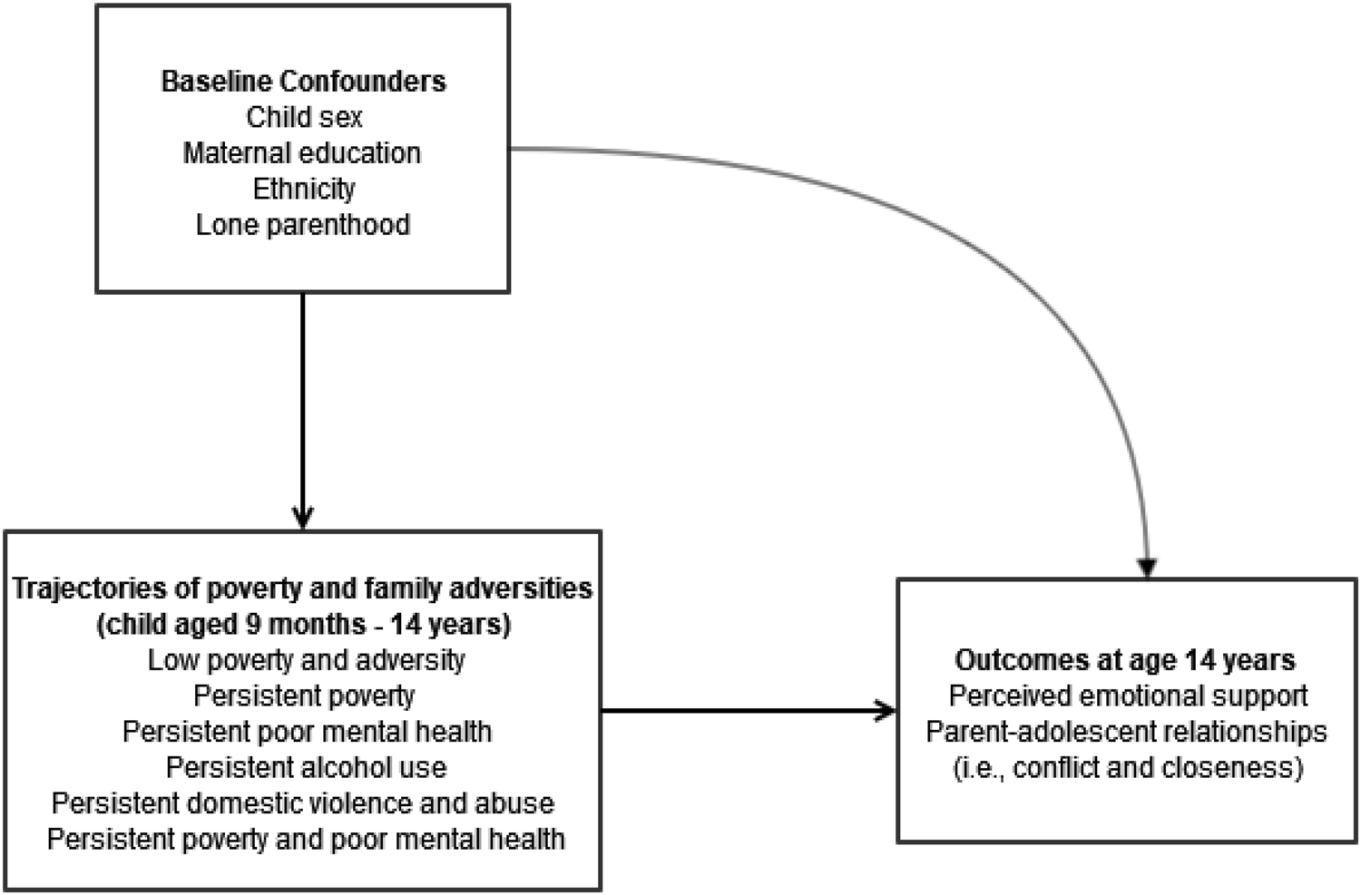
Directed acyclic graph (DAG) for the current study

We explored a range of potential confounding factors that were identified in previous analyses as being associated with the exposures and outcomes, guided by a directed acyclic graph (Fig. 1). These included child sex, maternal education (degree plus, diploma, A-levels, GCSE A-C, GCSE D-G, none), maternal ethnicity (white, mixed, Indian, Pakistani and Bangladeshi, black or Black British, or other ethnic groups) and lone parenthood when the child was aged 9 months.

### Statistical analysis

First, we characterised the exposure trajectories of poverty and family adversities using our previously developed group-based multi-trajectory models (age 9 months–14 years) with the *Traj* procedure in Stata (version 14.2) [16]. Then, we describe the sample characteristics. Percentages (%) were used to describe the prevalence of emotional support by baseline characteristics, with a focus on parental socio-economic status and by exposure trajectories. Differences in prevalence were examined using Pearson’s *Χ*^2^ test. Finally, we assessed the associations between the identified trajectories groups and outcomes at age 14 years (i.e., perceived emotional support and parent-adolescent relationships) using binary and ordinal logistic regression models, respectively, with 95% confidence intervals (CIs). Two models were built to test the effect of confounders: model 1 is the crude model to assess the association between predicted trajectory groups and each outcome, and model 2 is the adjusted model that controlled for potential confounding factors including, child sex, maternal education, ethnicity and lone parenthood. Both regression models included longitudinal weights that account for attrition, response bias and sampling design. To test the robustness of our analyses, we repeated the main results using multiple imputation by chained equation (*n* = 25) with results pooled using Rubin’s rules [20] to address missingness in the predictors and outcomes. Finally, we included interaction terms to assess whether the relationship between identified trajectory groups and outcomes varied by child’s sex. All statistical analyses were carried out using Stata (version 14.2).

## Results

### Study population characteristics

**Fig. 2:**
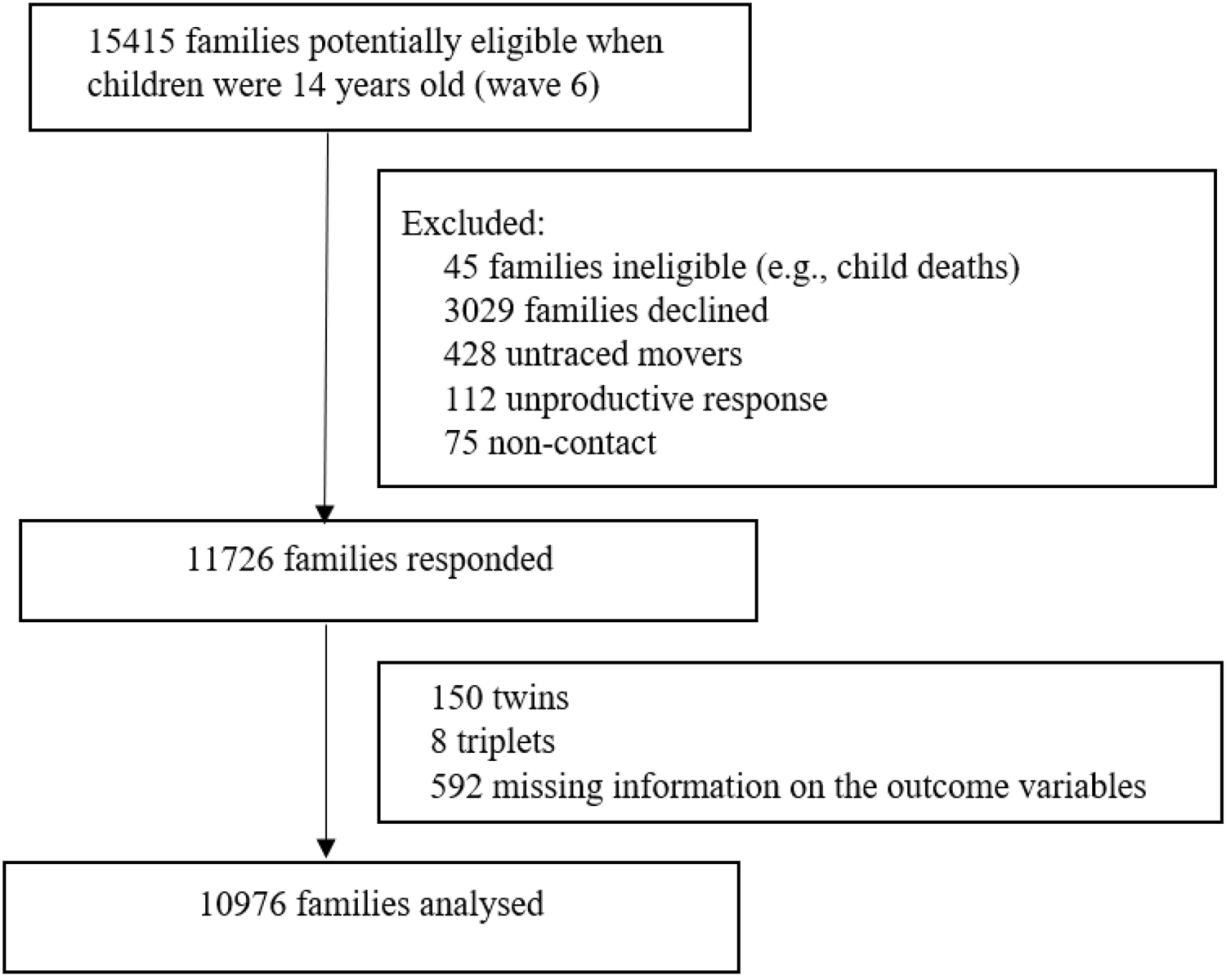
Study flow diagram showing inclusion and exclusion of cohort participant

Of the 15,415 families who were eligible at age 14 (wave 6), 10,976 families were analysed (Fig. 2). Table 1 shows the characteristics of the cohort participants by perceived emotional support (see imputed estimates in appendix, pp 2). A higher percentage of children who experienced low emotional support were in high adversity groups compared to those who experienced high emotional support. Differences based on socioeconomic status and ethnicity were also observed.

**Table 1:** Baseline characteristics and trajectories by perceived emotional support, observed data, weighted sample

	Perceived emotional support	
Characteristics	High (*n* = 9506)	Low (*n* = 1470)
Trajectories of poverty and family adversity		
Low poverty and adversity	4310 (45.3%)	530 (36.1%)
Persistent alcohol use	762 (8.0%)	92 (6.3%)
Persistent domestic violence and abuse	315 (3.3%)	59 (4.0%)
Persistent poor parental mental health	1115 (11.3%)	189 (12.9%)
Persistent poverty	2061 (21.6%)	370 (25.2%)
Persistent poverty and poor parental mental health	943 (9.9%)	230 (15.7%)
Child’s sex		
Boy	4508 (47.4%)	716 (48.7%)
Girl	4674(49.2%)	692 (47.1%)
Missing	324 (3.4%)	62 (4.2%)
Maternal education		
Degree plus	1884 (19.8%)	216 (14.7%)
Diploma	878 (9.2%)	113 (7.7%)
A-levels	913 (9.6%)	142 (9.7%)
GCSE A-C	2974 (31.3%)	478 (32.5%)
GCSE D-G	869 (9.1%)	152 (10.3%)
None	1647 (17.3%)	301 (20.4%)
Missing	341 (3.6%)	68 (4.6%)
Income Quintile		
Lowest	1492 (15.7%)	314 (21.4%)
Second	1520 (15.9%)	307 (20.9%)
Third	1934 (20.4%)	312 (21.2%)
Fourth	2263 (23.8%)	290 (19.7%)
Highest	2297 (24.2%)	247 (16.8%)
Maternal ethnicity		
White	7705 (81.1%)	1165 (79.3%)
Mixed	79 (0.8%)	14 (1.0%)
Indian	254 (2.7%)	36 (2.5%)
Pakistani and Bangladeshi	671 (7.1%)	116 (7.9%)
Black or Black British	289 (3.0%)	48 (3.3%)
Other ethnic groups	161 (1.7%)	28 (1.9%)
Missing	347 (3.7%)	63 (4.3%)

### Prevalence of low perceived emotional support

**Fig. 3:**
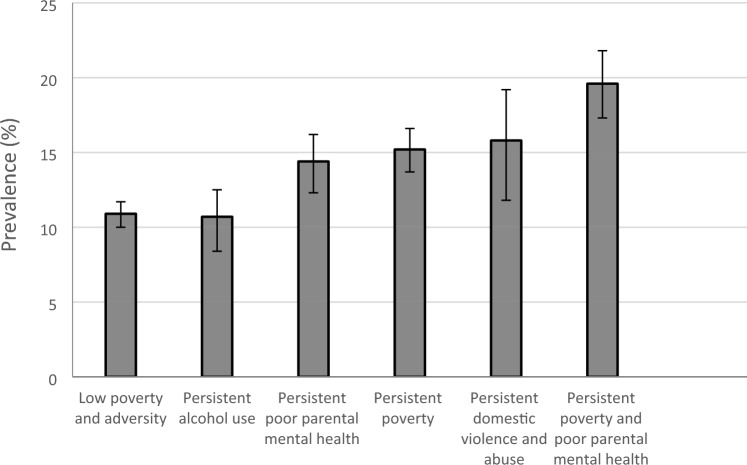
Prevalence of low emotional support by poverty and family adversity in the UK Millennium Cohort Study at age 14

**Fig. 4:**
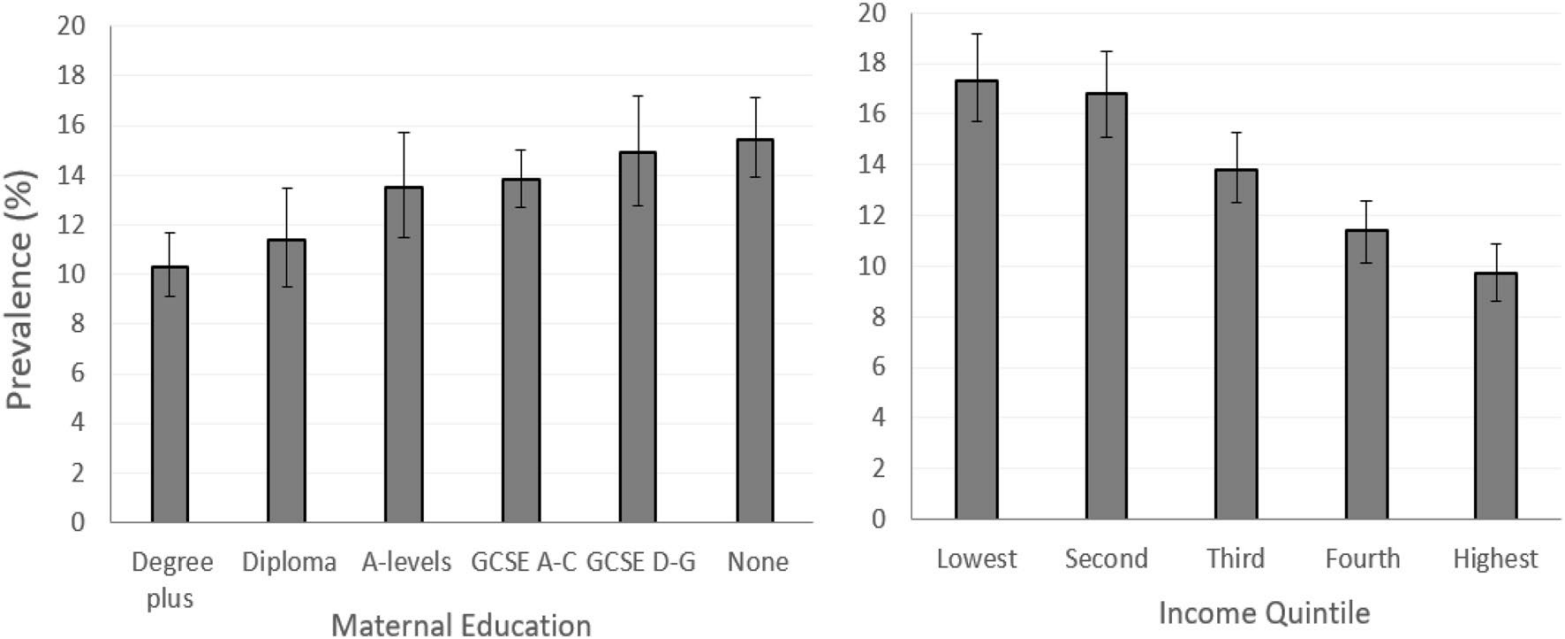
Prevalence of low emotional support by maternal education and income quintile in the UK Millennium Cohort Study at age 14

At age 14, the overall prevalence of low perceived emotional support was 13%. The prevalence of low perceived emotional support by adversity trajectories and baseline maternal education and household income, is shown in Figs. 3 and 4 respectively, and other baseline characteristics in supplementary Table 2 (pp 2). Disadvantaged children were more likely to experience low perceived emotional support. For example, the prevalence of low emotional support was 19.6% for children in the persistent poverty and poor parental mental health trajectory group compared to 10.9% in the low adversity and poverty trajectory group (Fig. 3).

We also found a monotonic relationship between education and income and level of emotional support, consistent with a social gradient. Children born to mothers with lower educational qualifications were more likely to experience low emotional support at age 14 years (education—degree plus: 10.3% vs. no qualifications: 15.4%). Those exposed to low family income were also found to be more likely to experience low support (income quintile—lowest: 17.3% vs. highest: 9.7%) (Fig. 4).

The outcomes at age 14 according to the six estimated trajectory groups are shown in Table 2. Children who were exposed to poverty and adversity, either singly or in combination, were more likely to experience low perceived emotional support. They also had a higher likelihood of not being attached to their parents and frequently exposed to family conflict. As expected, the prevalence of poor family relationships was higher in the co-occurrence of persistent adversity group. For example, 16% of children in the persistent poverty and poor parental mental health group reported being fairly close to their parents compared to 10% in the low poverty and low adversity group.

**Table 2:** Outcomes at age 14 according to the six estimated poverty and family adversity trajectory groups, observed data, weighted sample

Predicted family adversity and poverty trajectories						
Outcomes	Low poverty and adversity (*n* = 4840)	Persistent alcohol use (*n* = 854)	Persistent domestic violence and abuse (*n* = 374)	Persistent poor parental mental health (*n* = 1340)	Persistent poverty (*n* = 2431)	Persistent poverty and poor parental mental health (*n* = 1173)
Perceived emotional support						
Low	530 (10.7%)	92 (10.8%)	59 (15. 8%)	189 (14.5%)	370 (15.2%)	230 (19.6%)
High	4310 (89.3%)	762 (89.2%)	315 (84.2%)	1115 (85.5%)	2061 (84.8%)	943 (80.4%)
Closeness						
Extremely close	2354 (48.6%)	359 (42.0%)	169 (45.2%)	568 (43.6%)	1097 (45.1%)	467 (39.8%)
Very close	1962 (40.5%)	384 (45.9%)	145 (38.8%)	510 (39.1%)	878 (36.1%)	404 (34.4%)
Fairly close	474 (10.0%)	96 (11.2%)	51 (13.6%)	193 (14.8%)	294 (12.1%)	190 (16.2%)
Not very close	18 (0.4%)	5 (0.6%)	5 (1.3%)	8 (0.6%)	13 (0.5%)	20 (1.7%)
Missing	32 (0.7%)	10 (1.2%)	4 (1.1%)	25 (1.9%)	149 (6.1%)	92 (7.8%)
Conflict						
Hardly ever	1850 (38.2%)	313 (36.6%)	120 (32.0%)	385 (29.5%)	937 (38.5%)	345 (29.4%)
Less than once a week	1703 (35.2%)	287 (33.6%)	124 (33.2%)	434 (33.3%)	715 (29.4%)	277 (23.6%)
More than once a week	1005 (20.8%)	193 (22.6%)	95 (25.4%)	346 (26.5%)	427 (17.6%)	279 (23.7%)
Most days	238 (4.9%)	49 (5.7%)	31 (8.3%)	115 (8.8%)	184 (7.6%)	177 (15.1%)
Missing	44 (0.9%)	12 (1.4%)	4 (1.1%)	24 (1.8%)	168 (6.9	95 (8.1%)

### Associations of poverty and family adversity trajectories with perceived emotional support and parent-adolescent relationship

**Fig. 5:**
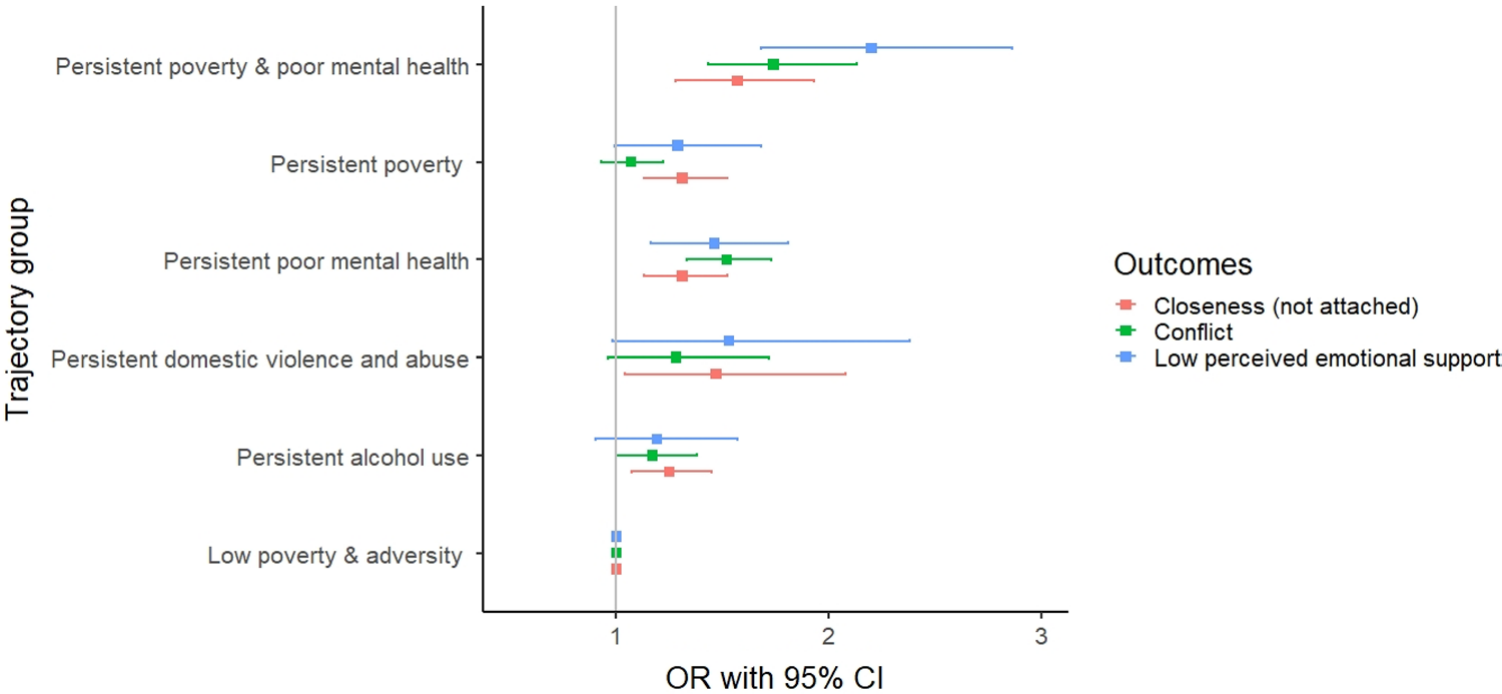
Associations of predicted family adversity and poverty trajectories and perceived adolescent outcomes at age 14 years in the UK Millennium Cohort Study

Figure 5 and Table 3 show the associations between predicted trajectory groups and perceived emotional support and quality of parent–adolescent relationship at age 14 years. Both the crude model (Model 1) and the adjusted model (Model 2) indicated that children in the persistent family adversity trajectory groups experienced higher odds of low emotional support and low-quality parent-adolescent relationship, when compared with those in the low poverty and adversity group. The associations were particularly strong for those exposed to both persistent poverty and poor parental mental health. For instance, when compared to children exposed to low poverty and adversity, those who experienced persistent poverty and poor parental mental health had a higher likelihood of experiencing low perceived emotional support (adjusted odds ratio 2·2; 95% CI 1·7–2·9), conflict (aOR 1.7; 95% CI 1·4–2·1), and not being attached to their parent (aOR 1·6; 95% CI 1·3–1·9). The adjustment for confounding factors did not result in any substantial attenuation of the associations when compared to the crude model (Table 3, Model 1 vs. Model 2). The regression analysis was also repeated analysis using imputed data (appendix pp 3), and the results were similar to the main results. The sensitivity analysis to determine if the association between the identified trajectory groups and outcomes varied by child’s sex showed no evidence of interaction (*p* > 0.05). We repeated the above models using Poisson regression and assessed the SPS-3 as a linear measure and found similar results (appendix pp 4).

**Table 3:** Associations of predicted family adversity and poverty trajectories and perceived adolescent outcomes at age 14 years in the UK Millennium Cohort Study (*n* = 10,548)

Odds ratio	Model^a^	Low poverty and adversity	Persistent alcohol use	Persistent domestic violence and abuse	Persistent poor parental mental health	Persistent poverty	Persistent poverty and parental poor mental health
Low perceived emotional support^b^	1	Ref	1.15 (0.87–1.50)	1.60 (1.04–2.41)	1.41 (1.16–1.72)	1.56 (1.32–1.84)	2.19 (1.79–2.72)
	2	Ref	1.19 (0.90–1.57)	1.53 (0.98–2.38)	1.46 (1.16–1.81)	1.29 (0.99–1.68)	2.20 (1.68–2.86)
Poor parent-adolescent Relationship^c^							
Argue/quarrel (conflict)	1	Ref	1.17 (0.99–1.36)	1.31 (0.97–1.74)	1.55 (1.37–1.76)	1.08 (0.96–1.22)	1.80 (1.52–2.76)
	2	Ref	1.17 (1.00–1.38)	1.28 (0.96–1.72)	1.52 (1.33–1.73)	1.07 (0.93–1.22)	1.74 (1.42–2.13)
Closeness (not attached)	1	Ref	1.26 (1.08–1.46)	1.46 (1.04–2.03)	1.25 (1.08–1.45)	1.19 (1.05–1.35)	1.46 (1.22–1.76)
	2	Ref	1.25 (1.07–1.45)	1.47 (1.04–2.08)	1.31(1.13–1.52)	1.31 (1.13–1.52)	1.57 (1.28–1.93)

^a^Model 1—crude model; Model 2—adjusted for child’s sex, maternal education, maternal ethnicity and lone parenthood

^b^Binary logistic regression

^c^Ordinal logistic regression

## Discussion

Using a large nationally representative cohort, we showed that more than 1 in 10 (13%) of young people in the UK experienced low perceived emotional support at age 14. Several key findings emerged from our analyses. Overall, we found that children of parents with low socioeconomic background were more likely to report low emotional support, consistent with a socio-economic gradient. Low perceived emotional support and poor family relationships in adolescence were more prevalent among socially disadvantaged children and children who experienced high levels of family adversity including parental mental illness, domestic violence and abuse and alcohol use throughout childhood and early adolescence. Over 10% of children experienced persistent poverty and persistent parental mental health in our trajectory analysis [16], and this combination of exposure was associated with more than twofold increased risk of low perceived emotional support and higher odds of low-quality parent–adolescent relationship at age 14 years.

Our estimate of low emotional support impacting 13% of young people in the UK is consistent with other studies [21]. To our knowledge, ours is the first longitudinal study to explore the relationship between early life course family-related risk factors and psychosocial needs in later adolescence. Few cross-sectional studies exist, but have mainly focused on the effects of single parental socioeconomic conditions such as family income and education [14], and surprisingly little is known about their social distribution [22]. In a cross-sectional study, Weyers et al. [14] found low social support to be more frequent among socio-economically disadvantaged people. Nonetheless, there is also some evidence from the recent Children’s Commissioner report suggesting that quality family relationships and strong social and emotional support can address social disadvantage in later life [7].

Previous studies of social and emotional support for young people have not looked at the impact of long-term patterns of multiple adversities on perceived quality of emotional support in adolescence [11]. Indeed, measures of emotional support are often used as mediating or interacting variables to assess protective or buffering effects against adverse health outcomes [23]. The evidence emerging from our longitudinal analysis shows that family emotional support is unevenly distributed across a wider social spectrum [24], which may have important implications for public policies and interventions.

Our analysis suggests that early-life social disadvantage and adversity are associated with lower levels of family support and poor family relationships in young adulthood. We further observed that each adversity (parental mental illness, domestic violence and abuse, alcohol use and poverty) in isolation was independently associated with an increased risk of low family support and a low-quality relationship. Children exposed to the combination of adversities, particularly persistent poverty and poor parental mental health were found to be at highest risk compared to those in the low adversity cluster group. This finding is consistent with the perspective that the availability of psychosocial resources, such as perceived emotional support, emerges from social conditions and other life experiences [25]. If true, then the exposure of children and young people to persistent adversity throughout childhood may be indicative of parents who experience higher levels of stress and are thus less able to provide emotional support to their children.

The potential mechanisms through which family-related stress impacts perceived psychological outcomes are well established [26]. Our longitudinal analyses add strength to the explanation that poverty and material deprivation may be potent causes of family stress [27], which may also erode the conditions under which emotional support can be provided to protect against stress. Numerous studies have demonstrated the connection between stressful life conditions such as income poverty and other family adversities including, family violence and mental health problems, such as depression and anxiety [16, 18, 28]. The co-occurrence of these mental health problems with economic disadvantage can greatly affect parenting behaviours and increase family conflict [26], which can negatively impact family support and the quality of family relationship [13, 26].

Further, the patterning of family support across socio-economic hierarchy can be explained in relation to family structure. Increasing evidence suggests that family structure and the context in which children grow up can play an important role in how social and economic disadvantage impacts young people’s psychosocial conditions [7]. While young people from two-parent households may experience some levels of stress caused by parental conflict and family violence [26, 29], several studies indicate that those from single-parent households are more likely to face psychological distress [7]. One possible reason for this is that single-parent households usually have fewer financial and emotional resources to meet their children’s needs, leading to increased stress and an unsupportive family atmosphere that negatively affects parent–child relationships [7]. Although this study did not directly investigate the various forms of family structure, unpacking and understanding the composition of contemporary families may be useful in developing effective family support programs and policies.

A key strength of the study is the use of a large and contemporary UK birth cohort, making the findings generalisable to the UK population regarding experiences of family adversities, emotional support, and quality of family relationships during adolescence. We also assume that our findings may apply to other high-income countries where poverty and other family adversities, such as parental mental health problems are prevalent. Additionally, a multi trajectory modelling technique was applied to predict multiple adversity exposures across childhood. This approach incorporates information on the timing and accumulation of experienced childhood adversities [16, 30].

However, a few limitations of this study should be noted. First, despite using validated tools and measures to assess our exposure trajectories and outcomes, we relied on self-reported information from cohort members and parents, which may be subject to reporting bias. Nevertheless, previous studies have found high internal consistency and reliability for our interest variables [18, 31], including our main outcome variable (i.e., perceived emotional support) [21]. Second, missing data are ubiquitous problems in longitudinal studies. We, however, repeated our analysis using imputation techniques to account for missing data. The sensitivity analysis comparing the main and imputed analyses showed similar results (see, appendix pp 3). Third, although the SPS-3 has been shown to be a reliable measure of perceived emotional support among adolescents [19], it is not specific to parents in the MCS. Nonetheless, the associations we found remain consistent for other outcomes specific to parents that we used in the study (i.e., closeness and conflict with parents). Lastly, although a wide range of information in our longitudinal data allowed us to adjust for potential confounders, unmeasured confounding factors such as genetics were not analysed due to data constraints.

Despite these limitations, our study makes a significant contribution to the literature on the social distribution and antecedents of emotional support by assessing significant links between social disadvantage during childhood, and levels of family support and quality of family relationships in adolescence. Drawing from a syndemic [32] and a life course perspective, our findings highlighted the importance of childhood and early adolescence and related family experiences for perceptions of emotional support in young adulthood.

From a public health perspective, our results corroborate other studies that advocate for a range of integrated responses to improve children’s life chances and break intergenerational cycles of adversity. This is likely to require a reduction in child poverty, actions to reduce other childhood adversities, and investment in both universal and targeted parenting support for families. Emotional support for young people within families does not spring from the ether. Our analysis suggests that poverty and family adversity are likely to have considerable impacts on parents’ ability to provide the necessary emotional support to children and young people. Child poverty, a major socioeconomic determinant of psychological and mental health [16, 18], is currently on the rise in the UK [16, 28]. Thus, policies aimed at improving family support for adolescents should also address easily modifiable determinants of child health such as child poverty in addition to prioritising more support for family mental health problems.

## Supplementary Information

Below is the link to the electronic supplementary material.

Supplementary file1 (DOCX 36 KB)

## Data Availability

All data used in this study are publicly available.
