# Supplementary material for: Impact of poverty and adversity on perceived family support in adolescence: findings from the UK Millennium Cohort Study
Source: Eur Child Adolesc Psychiatry. 2024 Feb 14;33(9):3123–32. doi: 10.1007/s00787-024-02389-8 (PMC11424735; doi:10.1007/s00787-024-02389-8)
Supplement: Supplementary file 1 — Supplementary file1 (DOCX 36 KB) [file 787_2024_2389_MOESM1_ESM.docx]

**Box 1.** Description of measurements assessed for trajectory exposures

- **Parental mental ill health (Child aged 9 months)** – Rutter Malaise Inventory (RMI)^16^ scale was used to assess parental mental ill health· A shortened 9-item self-completed version of the RMI measuring depression, anxiety and psychosomatic illness was used· The 9-item short form included items ‘feel tired most of the time’, ‘feel miserable or depressed’, ‘worried about things’, ‘often get into violent rage’ ‘suddenly become scared for no good reason’, ‘easily upset or irritated’, ‘constantly keyed up or jittery’, ‘every little thing gets on nerves and wears you out’, and ‘heart race like mad’· Scores from these items were summed, and we used a validated cut off for mental ill health [‘yes (scores >=4)/no’]·
- **Parental mental ill health (Child aged 3 to 14 years) –** Kessler 6 (K6)^17^ scale was used to assess parental mental ill health in the last 30 days asking the responders how often they felt depressed, hopeless, restless or fidgety, worthless, or that everything was an effort· Respondents answered on a five-point scale from 1(all the time) to 5 (none of the time)· We reversed and rescaled all items from 0 to 4 for analysis purposes, so that high scores indicate high levels of psychological distress· We used a validated cutoff widely used in previous studies [‘yes (scores >=6)/no’]
- **Frequent parental alcohol use (Child aged 9 months to 7 years) –** the main responder answered a question about their usual frequency of alcohol consumption (‘*Every day, 5-6 times per week, 3-4 times per week, 1-2 per week, 1-2 per month, less than once a month or never’*)·

Dichotomised: [every day and 5-6 times per week (Yes) vs· 3-4 per week/1-2 per week/ 1-2 per month/never (No)]

- **Frequent parental alcohol use (Child aged 11 to 14 years) –** the main responder answered a question about the usual frequency of alcohol consumption *(‘>=4 times per week, 2-3 times per week, 2-4 times per month, monthly or less, or never’)·*

Dichotomised: [4 or more times a week (Yes) vs· 2-3 per week/2-4 per month/ monthly or less/never (No)]

- **Domestic violence and abuse (Child aged 9 months to 14 years) –** the main responder was asked about the use of physical force by the partner in relationship (‘*Yes, No’*)
- **Poverty (Child aged 9 months to 14 years)** – relative income poverty^4^, defined as household equivalised income of less than 60% of national median household income equivalised according to the Organisation for Economic Co-operation and Development (OECD) household equivalence scale

**Supplementary Table 1.** Baseline characteristics and trajectories by perceived emotional support, observed data, imputed data

|  |  |  |
| --- | --- | --- |
|  | **Perceived emotional support** | |
| **Characteristics** | **High (n= 9,506)** | **Low (n=1,470)** |
| **Child's sex** |  |  |
| Boy | 49.2% | 51.1% |
| Girl | 50.8% | 48.9% |
| **Maternal education** |  |  |
| Degree plus | 20.5% | 15.4% |
| Diploma | 9.6% | 8.3% |
| A-levels | 9.9% | 10.3% |
| GCSE A-C | 32.4% | 33.5% |
| GCSE D-G | 9.5% | 10.9% |
| None | 18.0% | 21.5% |
| **Maternal ethnicity** |  |  |
| White | 84.2% | 82.7% |
| Mixed | 0.9% | 1.1% |
| Indian | 2.8% | 2.5% |
| Pakistani and Bangladeshi | 7.3% | 8.3% |
| Black or Black British | 3.0 % | 3.3% |
| Other ethnic groups | 1.7% | 2.1% |

Note: _Only percentages are presented for multiply imputed data as the_ *_ns_* _vary across the 25 imputed dataset_

**Supplementary Table 2.** Prevalence of low emotional support by child’s sex and maternal ethnicity in the UK Millennium Cohort Study at age 14

|  |  |  |
| --- | --- | --- |
| **Characteristics** | **Perceived emotional support (low)** | **P-value** |
| **Child's sex** |  | 0.138 |
| Boy | 13.7 (12.7-14.6) |  |
| Girl | 12.8 (12.0-13.8) |  |
| **Maternal ethnicity** |  | 0.661 |
| White | 13.1 (12.4-13.8) |  |
| Mixed | 15.0 (9.0-23.8) |  |
| Indian | 12.4 (9.1-16.7) |  |
| Pakistani and Bangladeshi | 14.7 (12.4-17.3) |  |
| Black or Black British | 14.2 (10.8-18.4) |  |
| Other ethnic groups | 14.8 (10.4-20.6) |  |

**Supplementary Table 3.** Associations of predicted family adversity and poverty trajectories and perceived adolescent outcomes at age 14 years in the UK Millennium Cohort Study, imputed

|  |  |  |  |  |  |  |
| --- | --- | --- | --- | --- | --- | --- |
| **Odds ratio** | Low poverty and adversity | Persistent alcohol use | Persistent domestic violence and abuse | Persistent poor parental mental health | Persistent poverty | Persistent poverty and parental poor mental health |
| **Low perceived emotional support**† | Ref. | 1.18 (0.89-1.56) | 1.58 (1.04-2.40) | 1.38 (1.12-1.69) | 1.49 (1.21-1.83) | 2.16 (1.72-2.72) |
| **Poor parent-adolescent Relationship**‡ |  |  |  |  |  |  |
| Argue/quarrel (conflict) | Ref. | 1.17 (1.00-1.38) | 1.28 (0.96-1.72) | 1.51 (1.32-1.72) | 1.06 (0.93-1.22) | 1.76 (1.43-2.15) |
| Closeness (not attached) | Ref. | 1.25 (1.07-1.45) | 1.47 (1.04-2.08) | 1.31 (1.13-1.52) | 1.31 (1.13-1.53) | 1.59 (1.29-1.95) |
|  |  |  |  |  |  |  |

Note: Model – adjusted for child's sex, maternal education, maternal ethnicity and lone parenthood; **†**Binary logistic regression; **‡**Ordinal logistic regression

**Supplementary Table 4.** Associations of predicted family adversity and poverty trajectories and perceived adolescent outcomes at age 14 years in the UK Millennium Cohort Study (full model)

|  |  |  |  |  |  |  |
| --- | --- | --- | --- | --- | --- | --- |
| **Odds ratio** | Low poverty and adversity | Persistent alcohol use | Persistent domestic violence and abuse | Persistent poor parental mental health | Persistent poverty | Persistent poverty and parental poor mental health |
| **Low perceived emotional support**† | Ref. | 1.21 (0.90-1.62) | 1.63 (1.03-2.58) | 1.57 (1.24-1.99) | 1.42 (1.07-1.87) | 2.69 (1.95-3.73) |
| **Poor parent-adolescent Relationship**‡ |  |  |  |  |  |  |
| Argue/quarrel (conflict) | Ref. | 1.15 (0.98-1.36) | 1.36 (0.99-1.87) | 1.46 (1.27-1.68) | 1.02 (0.92-1.25) | 1.44 (1.09-1.89) |
| Closeness (not attached) | Ref. | 1.31 (1.11-1.53) | 1.49 (1.01-2.20) | 1.25 (1.08-1.46) | 1.23 (1.03-1.47) | 1.53 (1.18-1.98) |
|  |  |  |  |  |  |  |

Note: Model – adjusted for baseline confounders (maternal age, child’s sex, maternal ethnicity) and time-variant confounders as repeated measures (maternal education and lone parenthood); **†**Binary logistic regression; **‡**Ordinal logistic regression

**Supplementary Table 5.** Associations of predicted family adversity and poverty trajectories and low perceived emotional support at age 14 years in the UK Millennium Cohort Study, poisson regression

|  |  |
| --- | --- |
| **Family adversity and poverty trajectories** | **RR (95% CI)** |
| Low poverty and adversity | Ref. |
| Persistent alcohol use | 1.16 (0.90-1.48) |
| Persistent domestic violence and abuse | 1.48 (1.04-2.11) |
| Persistent poor parental mental health | 1.32 (1.11-1.58) |
| Persistent poverty | 1.41 (1.18-1.68) |
| Persistent poverty and parental poor mental health | 2.03 (1.57-2.58) |

Note: Model – adjusted for child's sex, maternal education, maternal ethnicity and lone parenthood; RR- relative risk

**Supplementary Table 6.** Associations of predicted family adversity and poverty trajectories and perceived emotional support (score) at age 14 years in the UK Millennium Cohort Study, linear regression

|  |  |
| --- | --- |
| **Family adversity and poverty trajectories** | ***B* (SE)** |
| Low poverty and adversity | Ref. |
| Persistent alcohol use | -0.04 (0.04) |
| Persistent domestic violence and abuse | -0.19 (0.07)** |
| Persistent poor parental mental health | -0.15 (0.03)*** |
| Persistent poverty | -0.16 (0.03)*** |
| Persistent poverty and parental poor mental health | -0.33 (0.04)*** |

Note: Perceived emotional support (score): Low (3) – High (9); Significance level: *** p < 0.001; ** p < 0.01; * p < 0.05; Model – adjusted for child's sex, maternal education, maternal ethnicity and lone parenthood
